# Extracellular vesicles at the periodontal immune–regenerative interface: coupled mechanisms and translational challenges

**DOI:** 10.3389/fimmu.2026.1865803

**Published:** 2026-10-06

**Authors:** Ling Yan, Xinyi Sun, Yaping Chen, Lvhua Guo, Rong Liu

**Affiliations:** 1 Department of Prosthodontics, School and Hospital of Stomatology, Guangdong Engineering Research Center of Oral Restoration and Reconstruction & Guangzhou Key Laboratory of Basic and Applied Research of Oral Regenerative Medicine, Guangzhou Medical University, Guangzhou, China; 2 Department of Endodontics, School and Hospital of Stomatology, Guangdong Engineering Research Center of Oral Restoration and Reconstruction & Guangzhou Key Laboratory of Basic and Applied Research of Oral Regenerative Medicine, Guangzhou Medical University, Guangzhou, China; 3 Department of Oral Implantology, School and Hospital of Stomatology, Guangdong Engineering Research Center of Oral Restoration and Reconstruction & Guangzhou Key Laboratory of Basic and Applied Research of Oral Regenerative Medicine, Guangzhou Medical University, Guangzhou, China

**Keywords:** extracellular vesicle engineering, extracellular vesicles, immune regulation, microenvironment remodeling, periodontal regeneration, periodontitis, tissue-instructive signaling, translational challenges

## Abstract

Periodontitis is a chronic immune-inflammatory disease in which dysbiotic microbial communities and sustained host responses disrupt the gingiva, periodontal ligament (PDL), cementum, and alveolar bone. Because these tissues form an integrated attachment apparatus, effective regeneration requires coordinated control of inflammation and reconstruction of soft and mineralized tissues. Extracellular vesicles (EVs) function as intercellular mediators at this immune–regenerative interface by transferring proteins, lipids, and nucleic acids among immune, stromal, vascular, and bone-remodeling cells. This review synthesizes how EVs connect immune reprogramming, microenvironmental remodeling, and tissue-specific repair in periodontitis. Across experimental studies, EVs regulate macrophage activation and T-cell balance, modulate inflammatory, oxidative, vascular, and osteoclastogenic signaling, and influence the survival, migration, and differentiation of gingival, PDL, cementoblast, and osteoblast-lineage cells. Together, these actions link inflammatory resolution to the cellular processes required for gingival healing, alveolar bone formation, cementogenesis, and re-establishment of the PDL–cementum–bone interface. EV activity is shaped by donor-cell identity and physiological state, culture and separation procedures, molecular cargo, dose, recipient cell, and disease context; this context dependence explains why different EV preparations can produce reparative or pathogenic responses. Cargo engineering, surface modification, and biomaterial-assisted local delivery offer routes to enhance potency, cellular interaction, and retention within periodontal defects. Translation now depends on defining reproducible EV products, establishing mechanism-linked potency assays, standardizing dose and quality control, and evaluating safety and function in disease-relevant models. Current evidence is predominantly preclinical and indicates that process-defined EV preparations can link inflammatory resolution with tissue reconstruction as adjuncts to infection control and established periodontal regenerative procedures.

## Graphical Abstract

**Figure:**
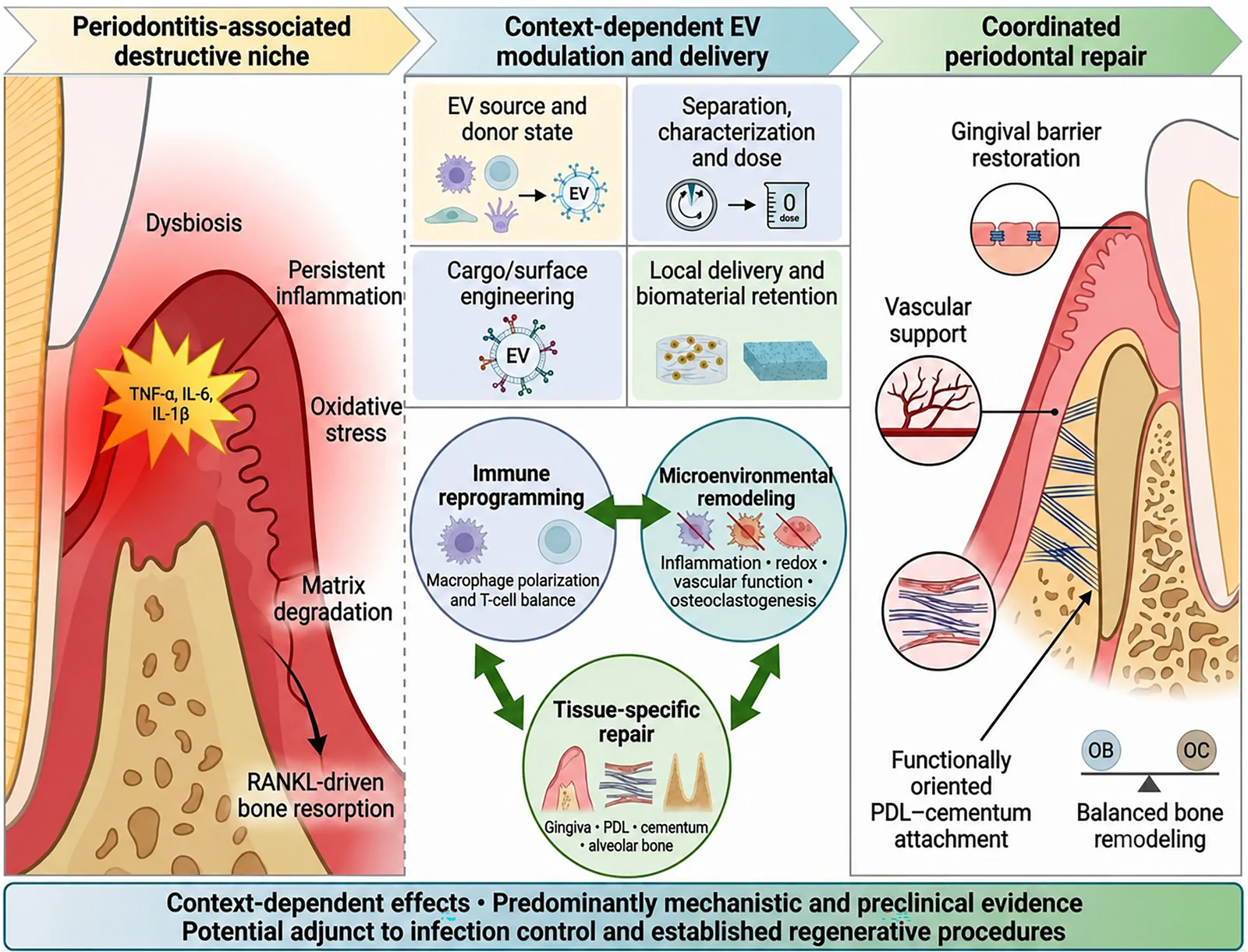
Created in BioRender. Guo, L. (2026). https://BioRender.com/md2tf2a.

## 1 Introduction

Periodontitis is a chronic inflammatory disease initiated by a dysbiotic subgingival biofilm and sustained by a dysregulated host response. Progressive destruction spans the gingival barrier, periodontal ligament (PDL), cementum, and alveolar bone, ultimately compromising tooth support (1, 2). These tissues function as an integrated attachment apparatus within a microbially challenged environment characterized by persistent cytokine production, oxidative stress, extracellular-matrix degradation, vascular dysfunction, and dysregulated bone remodeling. Periodontal regeneration therefore depends on coordinated reconstruction of alveolar bone, new cementum, functionally oriented PDL fibers, and gingival tissues, together with re-establishment of an immune and structural environment capable of maintaining the repaired attachment (3, 4).

The inflammatory response is closely integrated with the regenerative behavior of periodontal cells. Activated neutrophils, inflammatory macrophage states, and T helper 17 (Th17)-skewed adaptive responses contribute to cytokine production, oxidative injury, matrix degradation, and receptor activator of nuclear factor-κB ligand (RANKL)-dependent osteoclastogenesis, thereby linking immune dysregulation to connective-tissue and alveolar-bone destruction (5, 6). These signals also alter the proliferation, migration, and differentiation of resident stromal and progenitor cells. In inflammation-exposed periodontal ligament stem cells (PDLSCs), interactions among nuclear factor-κB (NF-κB), mitogen-activated protein kinase (MAPK), Wnt/β-catenin, and bone morphogenetic protein (BMP)/Smad signaling can restrict osteogenic differentiation (7, 8). Regenerative competence therefore depends on attenuation of destructive signaling and restoration of periodontal-cell responsiveness to instructive repair cues.

This immune–stromal interface is relevant beyond periodontology. Periodontitis provides a clinically accessible model of chronic, barrier-associated inflammation in which innate and adaptive immune activation, extracellular-matrix injury, vascular responses, and bone remodeling can be examined within the same tissue. The mechanisms discussed here therefore intersect with broader questions in osteoimmunology and chronic inflammatory disease: how persistent immune signals restrict progenitor-cell competence, how resolution restores responsiveness to repair cues, and how reconstructed tissue can reduce recurrent inflammatory activation.

Extracellular vesicles (EVs) have emerged as mediators of communication across this immune–stromal interface. By transporting surface-associated and luminal proteins, lipids, and nucleic acids, EVs convey donor-cell signals to immune, stromal, vascular, and bone-remodeling recipients and can be incorporated into local delivery systems. Their ability to transmit both inflammatory and reparative signals makes them relevant to the transition from periodontal destruction to tissue repair (9).

Previous reviews have catalogued EV sources, pathogenic roles, and regenerative applications in periodontitis (10–13). A remaining question is how EV-mediated immune regulation and changes in osteoclastogenic, oxidative, vascular, and matrix conditions interact with gingival, PDL, cementum, and alveolar-bone repair. Resolving these dependencies is necessary to distinguish isolated cellular responses from coordinated periodontal reconstruction.

To inform this narrative review, PubMed was searched from database inception to 22 August 2026 using combinations of terms related to periodontitis, periodontal regeneration, extracellular vesicles, and exosomes. Peer-reviewed studies were selected for their relevance to EV-mediated immune regulation, periodontal host-cell responses, tissue repair, EV engineering, delivery, and translational development; clinical-trial registries and regulatory sources were consulted for human studies and translational requirements. Evidence was synthesized around these mechanistic and translational themes, while EV nomenclature and the appraisal of separation, characterization, and functional attribution were guided by MISEV2023.

## 2 Extracellular vesicles: biogenesis, nomenclature, and functional properties

### 2.1 Biogenesis and nomenclature

Extracellular vesicles (EVs) are particles released by cells, delimited by a lipid bilayer, and incapable of independent replication. They comprise heterogeneous populations that differ in their biogenesis, size, molecular composition, and biological properties. EV populations can be conceptually distinguished according to their cellular site of biogenesis (**Figure 1A**). Exosomes originate from the endosomal system. During endosome maturation, inward budding of the limiting endosomal membrane generates intraluminal vesicles within multivesicular bodies. Multivesicular bodies may undergo lysosomal degradation or fuse with the plasma membrane, releasing their intraluminal vesicles into the extracellular space as exosomes. In contrast, ectosomes, often referred to as microvesicles, are released directly through outward budding and fission of the plasma membrane. Cells undergoing apoptosis can additionally release heterogeneous apoptotic EVs (9, 14).

**Figure 1:**
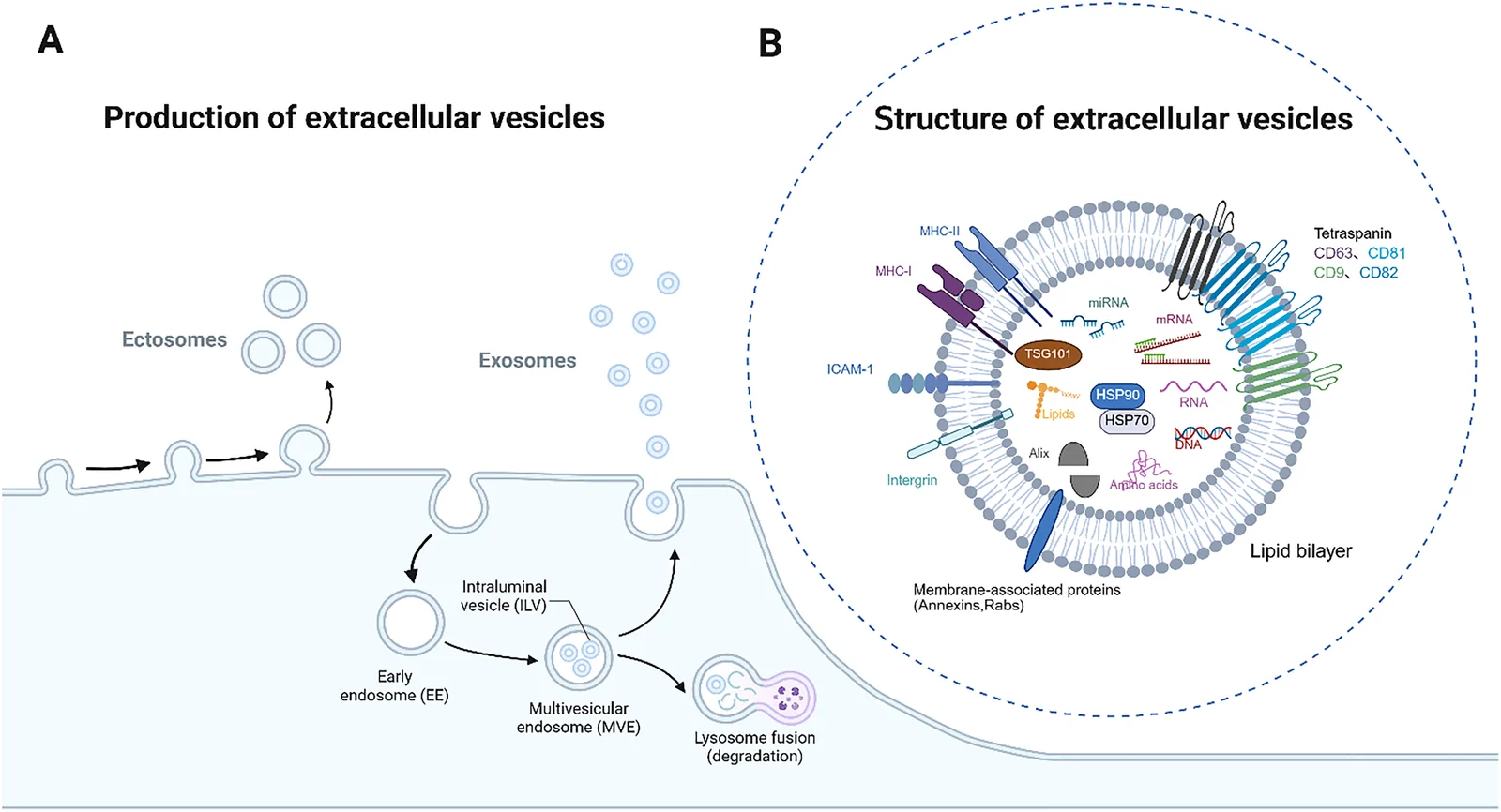
Biogenesis and representative composition of extracellular vesicles. **(A)** Exosomes arise from the endosomal pathway, whereas ectosomes bud directly from the plasma membrane. **(B)** EVs contain heterogeneous membrane-associated and luminal molecules; the illustrated components are representative. ALIX, ALG-2-interacting protein X; EE, early endosome; EV, extracellular vesicle; HSP, heat shock protein; ICAM-1, intercellular adhesion molecule 1; ILV, intraluminal vesicle; MHC, major histocompatibility complex; miRNA, microRNA; mRNA, messenger RNA; MVE, multivesicular endosome; TSG101, tumor susceptibility gene 101. Created in BioRender. Guo, L. (2026). https://BioRender.com/55nzacn.

Because exosomes, ectosomes, and apoptotic EVs overlap in size and molecular composition, routine separation and characterization generally define EV preparations operationally rather than establishing their biogenetic origin. Consistent with MISEV2023, “EV” is used as the default term when biogenesis remains unresolved, “sEV” denotes preparations enriched in small vesicles, commonly below approximately 200 nm, and “exosome” is reserved for vesicles whose endosomal origin is supported. When individual studies are discussed, their original terminology is retained, while the reported preparations are interpreted according to the available evidence for biogenesis, separation, and characterization (14).

### 2.2 Molecular composition and interactions with recipient cells

EVs can contain membrane and cytosolic proteins, lipids, metabolites, messenger RNAs, microRNAs, and other RNA species (**Figure 1B**). Their relative abundance and distribution vary with the cellular origin and physiological state of the producing cell, giving rise to substantial functional heterogeneity among EV populations (15).

Upon reaching recipient cells, EVs may initiate signaling through interactions between surface-associated molecules and cellular receptors. EV internalization provides an additional route through which vesicle-associated proteins, lipids, and nucleic acids may access intracellular compartments and influence gene expression, metabolism, or cell behavior. The lipid bilayer protects luminal molecules from extracellular degradation and allows multiple regulatory components to be transported within the same particle. Through these complementary modes of communication, EVs can coordinate responses across immune, stromal, vascular, and bone-remodeling cells within the periodontal microenvironment (9).

EVs constitute one component of the cellular secretome rather than an autonomous paracrine system. Conditioned medium also contains soluble cytokines, growth factors, extracellular proteins, and non-vesicular nucleic acid–protein or lipoprotein complexes, some of which may remain in EV-enriched preparations after separation. Biological activity observed with an EV-enriched preparation therefore does not by itself establish an EV-specific mechanism. Matched comparison of unfractionated conditioned medium, EV-enriched fractions, and EV-depleted fractions, together with disruption or functional blockade of the proposed EV-associated cargo, is required to distinguish vesicular signaling from soluble or co-isolated contributors.

Evidence from non-periodontal regenerative models indicates that EV-associated activity can reproduce or, under selected normalization conditions, exceed the activity of a broader secretome. In human placenta-derived MSC cultures, depletion of the exosome-enriched fraction reduced the proangiogenic effect of conditioned medium by approximately 60%, whereas the isolated fraction promoted endothelial tube formation to a degree comparable to unfractionated conditioned medium (16). In an adipose-tissue culture model, sEVs compared with the source conditioned medium at an equivalent protein concentration produced stronger effects on cell proliferation and migration and accelerated early adipose-tissue regeneration and angiogenesis; however, the two preparations produced similar tissue and vascular outcomes by week 12 (17). Together, these studies support a substantial EV-associated contribution in defined angiogenic and regenerative assays while showing that the relative contribution depends on dose normalization, endpoint, and time. Their relevance to periodontitis is therefore mechanistic: analogous matched-fraction experiments are still needed in dysbiotic periodontal models to determine the relative contributions of vesicular and soluble signals.

EV-based delivery occupies a distinct position between soluble biologics and living-cell therapy. Recombinant cytokines and other therapeutic proteins are compositionally defined and permit direct dose adjustment, but their short circulating half-lives, broad receptor distribution, and off-target effects often require repeated administration or molecular and carrier engineering (18). EVs protect and co-deliver membrane-associated and luminal cargos, whereas rapid systemic clearance, heterogeneous composition, and the lack of universally accepted potency units complicate dose–exposure relationships and batch comparability (19–21). Cell-based therapies can provide sustained and environmentally responsive secretion, but viability, biodistribution, donor- and culture-dependent variability, and manufacturing constraints make the administered biological dose more difficult to standardize (22). EVs therefore represent a distinct delivery modality rather than a uniformly superior one, exchanging the pharmacokinetic limitations of soluble proteins and the viability-dependent behavior of living cells for challenges in vesicle production, characterization, and potency control (23).

### 2.3 Plant-derived extracellular vesicle-like nanoparticles as a distinct particle class

Plant-derived extracellular vesicle-like nanoparticles (PELNs) constitute an operationally distinct particle class from mammalian cell-derived EVs. Many PELN preparations are recovered after juicing, homogenization, or mechanical disruption of plant tissues, procedures that do not establish extracellular release or a defined vesicle-biogenesis pathway. Their composition and biological activity may additionally reflect co-extracted lipids, proteins, polysaccharides, nucleic acids, and phytochemicals (24).

The most directly relevant periodontal evidence concerns ginger-derived preparations, which interact with *Porphyromonas gingivalis*, reduce virulence-associated activities, and attenuate periodontal destruction in preclinical models (25, 26). These findings position PELNs as a distinct antimicrobial and immunomodulatory platform for periodontal research.

## 3 Coupled EV regulation across periodontal host compartments

Periodontal repair depends on coordinated responses across immune, epithelial, stromal, vascular, and mineralized-tissue compartments (**Figure 2**). EV-mediated signaling participates in reciprocal interactions among immune reprogramming, microenvironmental remodeling, and tissue-specific repair: macrophage and T-cell responses reshape inflammatory, vascular, matrix, and osteoclastogenic conditions, while restoration of epithelial-barrier integrity limits microbial exposure and stromal, vascular, and mineralized-tissue repair feeds back on immune-cell recruitment and activation.

**Figure 2:**
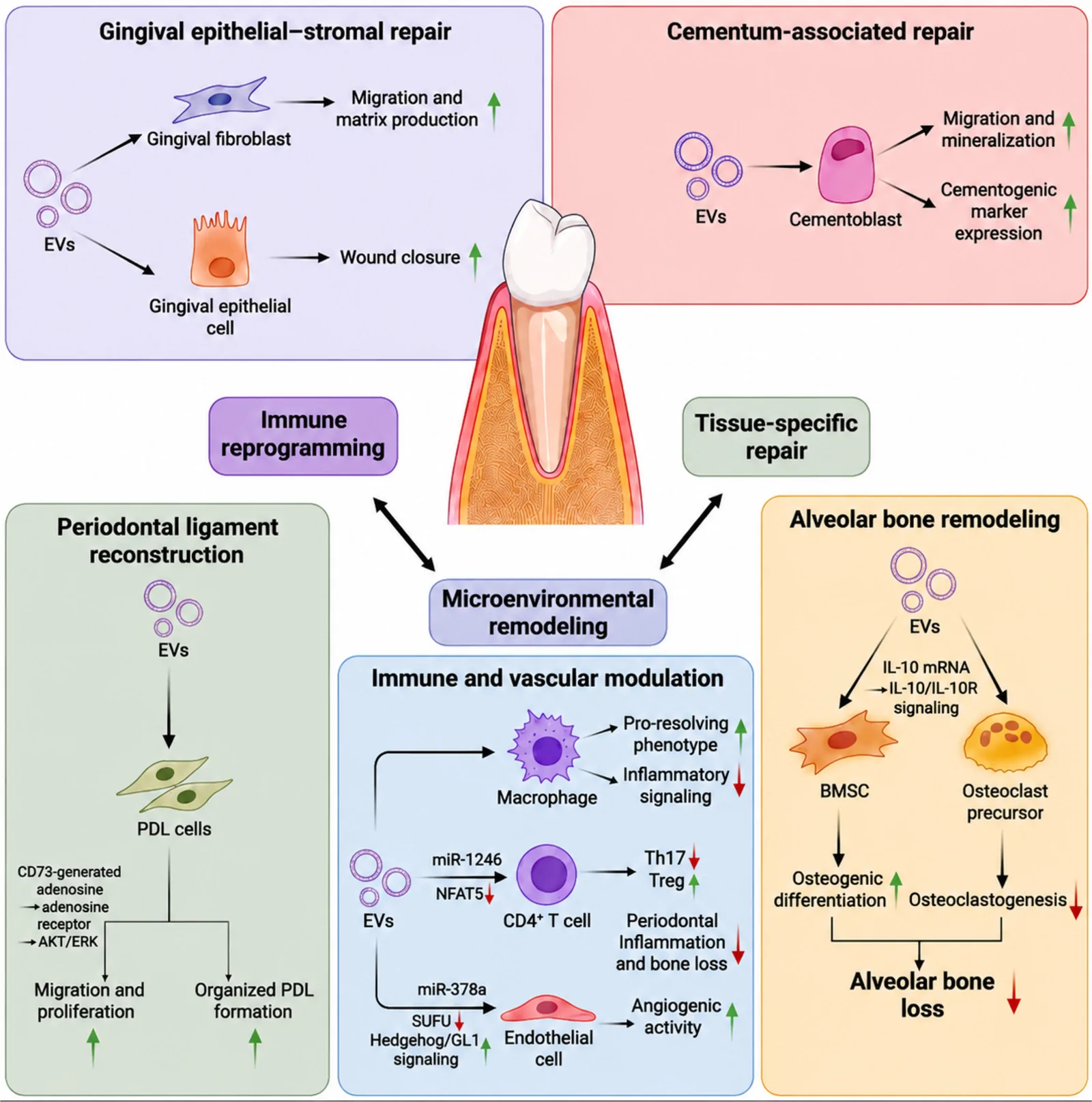
EV-mediated coordination of periodontal immune regulation and tissue repair. The pathways summarize findings obtained with distinct EV preparations. Green and red arrows indicate increased and decreased activity, respectively, and bidirectional arrows indicate reciprocal interactions. BMSC, bone marrow stromal cell; EV, extracellular vesicle; IL-10, interleukin-10; IL-10R, interleukin-10 receptor; NFAT5, nuclear factor of activated T cells 5; PDL, periodontal ligament; SUFU, suppressor of fused; Th17, T helper 17 cell; Treg, regulatory T cell. Created in BioRender. Guo, L. (2026) https://BioRender.com/eq5re8d.

### 3.1 Immune reprogramming as an upstream regulator

Immune regulation is a central determinant of the periodontal environment in which repair occurs. EV-mediated communication can reinforce or restrain destructive immune responses, thereby shaping the conditions for resident-cell repair.

#### 3.1.1 Macrophage activation and inflammatory resolution

Periodontal stromal-cell EVs can transmit either inflammatory or inflammation-resolving signals to macrophages through distinct molecular mechanisms. EVs released by lipopolysaccharide-stimulated PDLSCs enhanced pro-inflammatory macrophage activation through surface-associated DNA (27). A subsequent study identified miR-143-3p as an additional mediator enriched in EVs from inflammatory PDLSCs; by inhibiting phosphoinositide 3-kinase gamma (PI3Kγ)/AKT signaling and activating NF-κB, this miRNA promoted M1-like macrophage polarization and aggravated inflammation in experimental periodontitis (28). Gingival mesenchymal stromal cell (GMSC)-derived EVs instead promoted the resolution of macrophage inflammation. TNF-α preconditioning increased EV-associated CD73, expanded the CD206-positive macrophage population, and enhanced the reduction in osteoclast accumulation and alveolar bone loss in ligature-induced periodontitis (29). GMSC EVs also inhibited hypoxia-inducible factor-1 alpha (HIF-1α) activation and redirected inflammatory macrophage metabolism from glycolysis toward oxidative phosphorylation, supporting an anti-inflammatory transition *in vitro* and in rat periodontitis (30). These findings identify donor-cell conditioning and the resulting EV cargo as determinants of the macrophage response elicited by periodontal stromal-cell EVs.

Macrophage pyroptosis represents another EV-sensitive pathway supported by more than one periodontal study. PDLSC-derived EVs transferred miR-590-3p to macrophages, suppressed Toll-like receptor 4 (TLR4) and downstream NLRP3/caspase-1/GSDMD signaling, and reduced inflammatory injury in a murine ligature model; miR-590-3p inhibition and TLR4 rescue experiments supported the proposed mechanism (31). A later PDLSC small EV (sEV) study reported suppression of the same pyroptotic pathway and improved bone formation when the sEVs were incorporated into a thermosensitive chitosan hydrogel (32). The latter outcome reflects the combined activity of the EV preparation and its delivery matrix.

Macrophages can also act as EV donors, with effects that differ according to macrophage state. EVs from reparative M2-like macrophages transferred IL-10 mRNA to bone marrow stromal cells and osteoclast precursors, promoting osteogenic differentiation while suppressing osteoclast formation; IL-10 receptor blockade attenuated these effects, and EV administration reduced alveolar bone resorption in murine periodontitis (33). Conversely, apoptotic macrophage-derived vesicles enriched in miR-143-3p disrupted osteogenic-osteoclastic balance through IGFBP5 suppression and aggravated periodontal bone destruction (34).

Together, these studies place macrophage-centered EV communication at the interface between inflammatory control and periodontal bone remodeling. As recipients of stromal-cell EVs, macrophages translate pathological or reparative signals into changes in inflammatory and osteoclastogenic activity; as EV donors, they transmit their own functional state to bone-forming and bone-resorbing cells. This dual role makes macrophages a cellular link between EV-mediated immune regulation and alveolar bone remodeling.

#### 3.1.2 T-cell regulation and other immune populations

Changes in CD4^+^ T-cell effector and regulatory programs translate EV signals into altered periodontal inflammation and osteoclastogenic activity. PDLSC-derived EVs acted on the Th17/Treg axis through miR-155-5p/SIRT1 signaling, while EVs from three-dimensionally cultured dental pulp stem cells and bone marrow stromal cells independently implicated miR-1246 in restraining Th17 differentiation and enhancing regulatory responses (35–37). Gingival stromal cell-derived sEVs similarly suppressed CD4^+^ T-cell activation and IFN-γ production while promoting Treg formation (38). The resulting shift in T-cell activity reduced inflammatory and osteoclastogenic signaling within the periodontal microenvironment rather than ending at a change in lymphocyte phenotype.

Tregs also extend this communication outward by releasing EVs. EVs from retinoic acid-induced Tregs suppressed activated, IL-17A-expressing, and RANKL-expressing CD4^+^ T cells and reduced osteoclast-mediated alveolar bone loss in experimental periodontitis (39). Other Treg-derived EVs promoted PDLSC osteogenic differentiation and improved periodontal-defect repair, connecting adaptive immune regulation directly with resident-cell behavior (40). A related study associated the bone-protective effect of retinoic acid-induced Treg EVs with reduced macrophage infiltration and restoration of CD8^+^ T-cell abundance without expansion of IFN-γ- or RANKL-expressing CD8^+^ subsets (41). Treg-derived EVs therefore distribute regulatory signals across effector T cells, innate immune cells, and periodontal tissue-forming cells.

EV-mediated regulation also involves immune populations that connect innate sensing, adaptive polarization, and bone resorption. Dendritic cells link microbial recognition to T-cell differentiation: EVs released by *Porphyromonas gingivalis*-exposed dendritic cells propagated senescence-associated dysfunction, whereas immunoregulatory cargo-loaded dendritic-cell EVs suppressed recipient-cell maturation, shifted the Th17/Treg response toward regulation, and reduced osteoclastic bone loss (42, 43). B cells and plasma cells contribute to periodontal bone loss through BLyS/APRIL-dependent persistence and RANKL production, although their direct participation in periodontal EV signaling has not been established (44, 45).

Collectively, immune cells participate in EV communication as both recipients and donors. Stromal-cell EVs modify macrophage and T-cell programs, while EVs released by Tregs, macrophages, and dendritic cells distribute immune signals to additional immune populations and periodontal resident cells. These interactions connect immune-cell state with osteoclastogenic pressure, vascular and osteogenic support, and the conditions under which periodontal repair proceeds.

### 3.2 Gingiva: repair of the epithelial–stromal interface

The gingival epithelium and subjacent connective tissue form the first host barrier against the dysbiotic subgingival biofilm. During periodontitis, epithelial injury and disruption of intercellular junctions increase tissue exposure to microbial products, whereas gingival fibroblasts acquire inflammatory phenotypes that alter cytokine production, extracellular-matrix turnover, and communication with immune and bone-resorbing cells (46, 47). Restoration of this epithelial-stromal interface may reduce recurrent microbial stimulation and create a more permissive environment for repair in deeper periodontal compartments.

Current evidence indicates that EVs can influence both epithelial and fibroblast responses, although most studies have examined early wound-repair phenotypes rather than functional barrier restoration. EV preparations obtained from platelet lysate accelerated *in vitro* wound closure by human gingival keratinocytes and fibroblasts and altered the expression of matrix-remodeling genes, including FN1, COL1A1, and MMP1; these activities were retained after incorporation into a hyaluronic acid hydrogel (48). Inflammatory restraint may complement these direct effects on epithelial and stromal repair. Gingival mesenchymal stromal cell-derived sEVs carrying interleukin-1 receptor antagonist (IL-1RA) accelerated gingival wound closure in diabetic mice, whereas loss of IL-1RA reduced this effect (49). Similarly, adipose stromal cell-derived EVs improved gingival coverage and collagen deposition in a medication-related osteonecrosis model, and donor-cell IL-1RA knockdown attenuated EV-induced fibroblast migration, matrix production, and wound healing (50). Together, these findings indicate that EV-supported gingival repair may combine direct regulation of epithelial–stromal behavior with attenuation of inflammatory signals that impede wound closure.

EV signaling within the gingiva can also transmit pathological information. EVs released by fibroblasts from idiopathic gingival fibromatosis increased proliferation and reduced apoptosis in normal gingival fibroblasts, indicating the transfer of a disease-associated proliferative phenotype rather than a controlled regenerative response (51). Similarly, EVs from diabetic macrophages delivered miR-381-3p to gingival epithelial cells, suppressed the NR5A2–ATG7 autophagy axis, and enhanced inflammatory signaling *in vitro* (52). These findings indicate that EVs released from stressed or diseased gingival cells can propagate inflammatory and senescence-associated signals, thereby impairing epithelial–stromal repair and sustaining a tissue environment unfavorable for periodontal regeneration.

Evidence therefore supports EV effects on gingival-cell migration, survival, matrix-related activity, and inflammatory signaling more strongly than restoration of a functional epithelial barrier.

### 3.3 Periodontal ligament: restoring progenitor competence and functional attachment

The periodontal ligament (PDL) is a highly organized, non-mineralized connective tissue that anchors the tooth root to alveolar bone, distributes mechanical load, and contains resident progenitor populations involved in periodontal homeostasis and repair. During periodontitis, sustained exposure to microbial products, inflammatory cytokines, oxidative stress, and matrix-degrading enzymes can alter the immunoregulatory properties of PDL cells and restrict the regenerative competence of periodontal ligament stem cells (PDLSCs). Successful PDL regeneration therefore requires more than increased cell proliferation or osteogenic differentiation. Functional PDL restoration is defined by formation of a spatially organized ligament in which collagen fibers insert into newly formed cementum and alveolar bone, maintain an appropriate ligament space, and avoid ankylosis (3, 4).

Several *in vitro* studies indicate that EVs can relieve inflammatory constraints on PDLSCs. PDLSC-derived EVs suppressed both basal and lipopolysaccharide-induced NF-κB activity in recipient PDLSCs, potentially through interactions involving TLR4 and PI3K/AKT signaling. Notably, these EVs altered the expression of osteogenesis-related genes but did not directly increase mineralization, indicating that attenuation of inflammatory signaling does not necessarily produce an osteogenic response (53). Gingival mesenchymal stromal cell-derived EVs similarly reduced LPS-induced inflammatory responses in PDLSCs, accompanied by inhibition of NF-κB signaling and Wnt5a expression (54). Further work showed that these EVs inhibited *Porphyromonas gingivalis* LPS-induced NF-κB activation while restoring Wnt/β-catenin signaling and osteogenic-marker expression. Pharmacological inhibition of Wnt/β-catenin signaling abolished the suppressive effect on NF-κB, supporting functional coupling between inflammatory control and regenerative signaling in PDLSCs (55). These studies establish pathway-level effects but do not identify the individual EV-associated molecules responsible for this reciprocal regulation.

In addition to alleviating inflammatory constraints, EVs can provide tissue-instructive signals that directly influence PDLSC lineage activity. EV preparations derived from osteogenically conditioned stem cells from human exfoliated deciduous teeth (SHED) enhanced PDLSC mineralization and activated BMP/Smad and Wnt/β-catenin signaling *in vitro*. Wnt3a and BMP2 were detected in the EV preparations, and donor-cell knockdown of either factor partially reduced the osteogenic response of recipient PDLSCs, providing functional evidence that these EV-associated proteins contributed to the observed effect (56). SHED- and dental pulp stem cell-derived sEVs have also been compared directly. Both promoted PDLSC proliferation, migration, and osteogenic differentiation *in vitro*, whereas SHED-derived sEVs exhibited greater particle productivity and stronger osteogenic effects under the tested conditions (57). However, this comparison did not identify the molecular cargo responsible for the difference or determine whether the observed mineralization translated into PDL-specific tissue formation.

Histological evidence of PDL organization was provided by a surgically created rat intrabony-defect model, in which mesenchymal stromal cell-derived exosomes delivered in a collagen sponge increased new bone and PDL formation. Obliquely oriented fibers extended between the root surface and newly formed bone, and ankylosis was not observed (58).

Collectively, EVs support PDL repair through two complementary processes: relief of inflammatory constraints on PDLSC function and provision of signals that promote cell recruitment, survival, and lineage-related activity. Whether these effects culminate in functional PDL reconstruction under persistent inflammatory and microbial challenge remains unresolved.

### 3.4 Vascular compartment: angiogenic signaling in periodontal repair

The periodontal vasculature supports tissue repair by supplying oxygen and nutrients and by regulating leukocyte recruitment, vascular permeability, and extracellular-matrix remodeling. These functions become intertwined in periodontitis, where angiogenesis occurs within an inflamed and structurally disrupted tissue environment (59).

Inflammatory conditions alter EV-mediated communication between periodontal stromal and endothelial cells. Inflamed PDLSCs released more EVs and enhanced human umbilical vein endothelial cell tube formation through EV-associated VEGFA; reduced miR-17-5p expression in the donor PDLSCs relieved repression of VEGFA and increased its incorporation into the angiogenic response (60). Similarly, EVs from periodontitis-compromised dental pulp stem cells were enriched in miR-378a and promoted endothelial-cell proliferation, migration, and tube formation by suppressing Sufu and activating Hedgehog/Gli1 signaling (61). These pathways show how disease-associated stromal cells amplify angiogenic signaling within inflamed periodontal tissues.

Evidence connecting EV-mediated angiogenesis with tissue repair has been obtained using SHED-derived EVs. These EVs enhanced endothelial angiogenic activity and bone marrow stromal cell osteogenic differentiation through AMPK-associated signaling *in vitro* and, when delivered with β-tricalcium phosphate in rat periodontal bone defects, increased vascular-marker expression and new bone formation (62). The concurrent vascular and bone-forming responses support a role for EV-mediated angiogenic signaling during defect repair. However, the current studies primarily measure endothelial proliferation, tube formation, and vascular-marker expression; whether these responses generate stable, perfused vessels and normalize vascular permeability or leukocyte trafficking has not been determined.

### 3.5 Alveolar bone: restoring coupled bone remodeling

Alveolar bone is continuously remodeled through the coordinated activities of osteoblast-lineage cells, osteocytes, and osteoclasts. In periodontitis, inflammatory cytokines, oxidative stress, and RANKL-dominant signaling increase osteoclast formation and activity while impairing the differentiation and matrix-producing capacity of osteoblast-lineage progenitors. EV-mediated preservation or restoration of alveolar bone may therefore arise through increased bone formation, reduced pathological resorption, or both (5, 6).

#### 3.5.1 Osteoblast-lineage support and bone formation

Dental and non-dental stromal cell-derived EVs have increased osteogenic-marker expression, alkaline phosphatase activity, and mineral deposition in PDLSCs, bone marrow stromal cells, and osteoblast-lineage cells across multiple experimental systems (33, 56, 63, 64). These effects have been associated with BMP/Smad, Wnt/β-catenin, PI3K/AKT, AMPK, and cargo-dependent pathways. Most findings, however, derive from *in vitro* differentiation assays or non-periodontal bone-defect models and demonstrate osteogenic potential rather than formation of structurally and functionally integrated alveolar bone.

Preclinical studies indicate that osteogenic responses can occur alongside vascular and immune changes. SHED-derived EVs combined with β-tricalcium phosphate increased vascular-marker expression and new bone formation in rat periodontal defects, whereas hydrogel-delivered BMSC sEVs reduced inflammatory infiltration, collagen destruction, osteoclast numbers, and alveolar bone loss while supporting PDL-cell osteogenic activity (62, 65). EVs from low-intensity-pulsed-ultrasound-stimulated apical papilla stem cells and from LPS-preconditioned dental follicle cells produced similar combinations of anti-inflammatory, cell-protective, and mineralized-tissue effects in periodontal models (66, 67). These studies indicate that changes in mineralized tissue can reflect concurrent effects on bone-forming cells, vascular responses, inflammation, and osteoclast activity.

#### 3.5.2 Osteoclast regulation and protection against inflammatory bone loss

EVs from TNF-α-preconditioned GMSCs were enriched in miR-1260b, which suppressed Wnt5a-dependent RANKL expression in PDL cells. In experimental periodontitis, the same EV preparation reduced TRAP-positive osteoclast numbers and alveolar bone resorption (29). Reparative M2-like macrophage-derived EVs likewise coupled IL-10 receptor-dependent promotion of stromal-cell osteogenesis with inhibition of osteoclast differentiation and reduced alveolar bone resorption *in vivo* (33). Cargo detection, recipient-cell transfer, receptor blockade, and *in vivo* validation provide comparatively strong support for the involvement of the EV-associated IL-10 mRNA–IL-10 receptor axis.

Osteoclastogenesis is not uniformly suppressed by EV treatment; under disease-associated donor states and during physiological remodeling, EV signaling can instead promote osteoclast differentiation. Macrophage-derived apoptotic vesicles carrying miR-143-3p disrupted osteogenic–osteoclastic balance through IGFBP5 suppression and aggravated periodontal bone destruction (34). EVs derived from human PDL cells promoted osteoclast differentiation *in vitro* while shifting macrophages toward an M2-like phenotype, demonstrating that an M2-associated response does not necessarily reduce resorption (68). Mechanical loading also altered PDLSC-derived EVs: mechanically stimulated PDLSCs released annexin A3-enriched EVs that enhanced internalization, activated ERK signaling, and promoted osteoclast differentiation during experimental tooth movement (69). In that setting, osteoclastogenesis supports mechanically induced remodeling rather than inflammatory periodontal destruction.

Overall, EVs can shift alveolar bone remodeling through direct effects on osteoblast- and osteoclast-lineage cells and through upstream regulation of macrophages, PDL cells, and vascular responses. NF-κB- and RANKL-dependent signaling connect these compartments: persistent inflammatory activation promotes osteoclastogenesis and restricts osteogenic differentiation, whereas immune resolution reduces resorptive pressure and permits bone-forming cells to respond to regenerative cues. Determining whether this rebalanced remodeling contributes to attachment reconstruction requires simultaneous assessment of bone formation and resorption together with the PDL–cementum interface.

### 3.6 Cementum: reconstruction of the root attachment surface

Cementum provides the mineralized root surface into which periodontal ligament fibers are inserted and is therefore indispensable for re-establishing functional tooth attachment. Its regeneration differs from generalized mineralized-tissue formation: an increase in calcium deposition or osteogenic markers does not necessarily indicate the formation of structurally organized cementum capable of supporting periodontal ligament attachment (70). Compared with the evidence concerning alveolar bone and periodontal ligament, studies directly examining EV-mediated cementum repair remain limited.

Macrophage-derived EVs can influence cementoblast mineralization. M1-polarized macrophages and their EVs suppressed cementoblast mineralization, whereas M2-polarized macrophages and their EVs enhanced it *in vitro*. In models of mechanically induced tooth movement and local LPS exposure, an increased M1/M2 ratio was accompanied by reduced cementoblast mineralization and root resorption; administration of M1- and M2-derived EVs produced corresponding inhibitory and stimulatory effects on cementoblast mineralization *in vivo* (71). Human PDLSC-derived EVs also promoted OCCM-30 cementoblast proliferation, migration, mineral deposition, and the expression of cementogenesis-associated factors *in vitro* through PI3K/AKT signaling (72). A specific EV cargo mechanism was identified using Ckip-1-silenced M2-like macrophages. EVs from these cells transferred let-7f-5p to cementoblasts, where it targeted Ckip-1 and activated PGC-1α-dependent mitochondrial biogenesis. The EVs restored mineralization in *P. gingivalis*-exposed cementoblasts and rescued cementum-like tissue formation in an ectopic model in which cementoblast-loaded gelatin sponges were implanted subcutaneously in nude mice (73).

These studies establish cementoblasts as EV-responsive cells and identify macrophage polarization, PDLSC-derived signaling, and the let-7f-5p–Ckip-1–PGC-1α axis as regulators of cementoblast mineralization. Among them, Huang et al. demonstrated cementum-like tissue formation *in vivo*, but in an ectopic implantation model rather than on a periodontal root surface. Direct evidence that EV treatment generates new root-surface cementum with inserted, appropriately oriented PDL fibers remains unavailable.

Representative studies across these periodontal compartments, including their EV sources, experimental models, principal outcomes, and methodological characteristics, are summarized in **Table 1**.

**Table 1:** Evidence map of representative mechanistic and preclinical studies of extracellular vesicles in periodontal immune regulation and tissue repair.

Biological process	EV source	Experimental model	EV isolation	EV characterization	Cargo or signaling pathway	Main finding	Ref.
Immune and inflammatory regulation	LPS-preconditioned human PDLSCs	*In vitro* macrophage-polarization assays	Differential centrifugation, filtration, and UC	NTA; WB	DNase-sensitive EV-associated nucleic acids	Induced pro-inflammatory macrophage activation	(27)
Immune and inflammatory regulation	Human PDLSCs cultured under basal or LPS-stimulated conditions	Human peripheral immune-cell profiling and *in vitro* CD4^+^ T-cell assays	Sequential centrifugation and UC	TEM; WB	miR-155-5p–SIRT1	Regulated the Th17/Treg balance according to the inflammatory state of the EV-producing PDLSCs	(35)
Immune and inflammatory regulation	Microcarrier-expanded human PDLSCs	*In vitro* PDLSC NF-κB reporter and osteogenic assays	Differential UC	NTA; WB	TLR4 interaction and PI3K/AKT–GSK3β signaling	Suppressed basal and LPS-induced NF-κB activity in PDLSCs	(53)
Immune and inflammatory regulation	TNF-α-preconditioned human GMSCs	*In vitro* macrophage and PDLC assays; mouse ligature-induced periodontitis	Filtration, concentration, and phosphatidylserine-affinity isolation	TEM; NTA; WB	CD73-dependent macrophage regulation; miR-1260b–Wnt5a–RANKL signaling	Promoted M2-like macrophage responses and reduced RANKL signaling, osteoclast numbers, and alveolar bone resorption	(29)
Immune and inflammatory regulation	Human DPSCs cultured in two-dimensional and three-dimensional systems	*In vitro* T-cell assays; mouse ligature-induced periodontitis	Sequential centrifugation and UC	TEM; NTA; flow cytometry; WB	miR-1246–NFAT5 signaling	Three-dimensional culture enhanced EV-mediated regulation of the Th17/Treg balance and reduced alveolar bone loss	(36)
Immune and inflammatory regulation	Human PDLSCs	LPS- and ATP-stimulated macrophages; mouse ligature-induced periodontitis	Sequential centrifugation and UC	TEM; NTA; WB	miR-590-3p–TLR4–NLRP3/caspase-1/GSDMD signaling	Reduced macrophage pyroptosis and periodontal inflammatory injury	(31)
Immune and inflammatory regulation	Human GMSCs	P. gingivalis LPS-stimulated PDLSCs	Sequential centrifugation, filtration, and UC	TEM; NTA; WB	Wnt/β-catenin–NF-κB cross-regulation	Promoted PDLSC osteogenic differentiation and suppressed NF-κB activation under inflammatory conditions	(55)
Immune and inflammatory regulation	Human GMSCs	THP-1 macrophages; rat periodontitis	—	TEM; NTA; WB	HIF-1α inhibition and glycolysis-to-OXPHOS metabolic reprogramming	Shifted inflammatory macrophages toward an M2-like metabolic and phenotypic state	(30)
Immune and inflammatory regulation	Human PDLSCs	Macrophage/PDLSC assays; rat periodontal defect	UC	TEM; NTA; WB	NF-κB–NLRP3–caspase-1–GSDMD signaling	Reduced macrophage pyroptosis and promoted alveolar bone regeneration when delivered in hydrogel	(32)
Immune and inflammatory regulation	Three-dimensionally cultured human DPSCs producing VNN2-enriched EVs	Human gingival-tissue analysis; LPS-stimulated PDLSCs; rat periodontitis	Sequential centrifugation and UC	TEM; NTA; WB	EV-associated VNN2 signaling in PDLSCs	Increased inflammatory signaling, impaired PDLSC osteogenic differentiation, and aggravated alveolar bone loss	(74)
Immune and inflammatory regulation	Human maxillary bone marrow-derived MSCs	Patient-derived CD4^+^ T cells; mouse ligature-induced periodontitis	UF and polymer precipitation	TEM; NTA; flow cytometry	miR-1246–ACE2–YAP1 signaling	Reduced Th17 predominance and alveolar bone loss	(37)
Periodontal ligament and osteogenic repair	Immortalized human embryonic stem-cell-derived MSCs	*In vitro* PDL-cell assays; collagen-sponge treatment of rat intrabony defects	Tangential-flow filtration	Particle-size, density, and EV-protein analyses	CD73–adenosine receptor–AKT/ERK signaling	Increased PDL-cell migration and proliferation and promoted bone formation and organized PDL formation in rat defects	(58)
Periodontal ligament and osteogenic repair	Osteogenically conditioned SHED	*In vitro* PDLSC osteogenic assays	Sequential centrifugation and UC	TEM; NTA; WB	Wnt3a/BMP2 and Wnt/β-catenin–BMP/Smad signaling	Enhanced PDLSC osteogenic differentiation	(56)
Periodontal ligament and osteogenic repair	P2X7R-engineered human PDLSCs	Inflammation-compromised PDLSCs	Polymer precipitation	TEM; NTA; WB	miR-3679-5p, miR-6747-5p, and miR-6515-5p–GREM1 signaling	Restored osteogenic differentiation in inflammation-compromised PDLSCs	(75)
Periodontal ligament and osteogenic repair	LIPUS-stimulated SCAP	*In vitro* PDLC assays; mouse inflammatory alveolar bone loss	Sequential centrifugation, filtration, and UC	TEM; NTA; WB	nSMase2-dependent EV release and miR-935 signaling	Increased EV release, enhanced PDLC osteogenic responses, and reduced inflammatory alveolar bone loss	(66)
Periodontal ligament and osteogenic repair	Dental follicle stem cells producing miR-423-5p-enriched sEVs	*In vitro* PDLC assays; rat periodontal defects	UF and membrane-affinity chromatography followed by post-loading UF	TEM; nano-flow cytometry; WB; zeta-potential analysis; cargo qPCR	miR-423-5p–PLCB1–Wnt/β-catenin signaling	Enhanced early osteogenesis and formation of a native-like cementum–PDL–alveolar bone complex	(76)
Vascular support	Periodontitis-compromised and healthy human DPSCs	*In vitro* endothelial-cell proliferation, migration, and tube-formation assays	Sequential centrifugation and UC	TEM; NTA; WB	miR-378a–SUFU–Hedgehog/GLI1–VEGF signaling	EVs from periodontitis-compromised DPSCs enhanced endothelial proliferation, migration, and tube formation	(61)
Alveolar bone remodeling	M2-like macrophages	*In vitro* BMSC and osteoclast assays; mouse periodontitis	Filtration and UC	TEM; DLS; WB	EV-associated IL-10 mRNA–IL-10R signaling	Promoted BMSC osteogenesis, inhibited osteoclast differentiation, and reduced alveolar bone resorption	(33)
Cementum-associated repair	CKIP-1-silenced M2-like macrophages	*In vitro* cementoblast assays; ectopic implantation in nude mice	Sequential centrifugation, filtration, and UC	TEM; DLS; WB	let-7f-5p–CKIP-1–PGC-1α signaling	Restored cementoblast mineralization and cementum-like tissue formation in an ectopic implantation model	(73)

ACE2, angiotensin-converting enzyme 2; BMSC, bone marrow stromal cell; DLS, dynamic light scattering; DPSC, dental pulp stem cell; EV, extracellular vesicle; GMSC, gingival mesenchymal stromal cell; LIPUS, low-intensity pulsed ultrasound; NTA, nanoparticle tracking analysis; OXPHOS, oxidative phosphorylation; PDLC, periodontal ligament cell; PDLSC, periodontal ligament stem cell; SCAP, stem cells from the apical papilla; sEV, small extracellular vesicle; SHED, stem cells from human exfoliated deciduous teeth; TEM, transmission electron microscopy; UC, ultracentrifugation; UF, ultrafiltration; WB, western blotting.

## 4 Engineering and delivery strategies for periodontal EV therapy

For periodontal applications, EV engineering aims to enhance biological potency, tissue targeting, and local retention. The principal approaches include cargo engineering, surface modification, and biomaterial-assisted delivery (77).

### 4.1 Cargo engineering and donor-cell conditioning

Donor-cell manipulation modifies EV cargo during vesicle biogenesis. Three-dimensional culture of dental pulp stem cells (DPSCs) increased EV miR-1246 abundance and enhanced regulation of Th17/Treg responses, whereas VNN2-enriched EVs from another three-dimensional DPSC system intensified inflammatory signaling in PDLSCs, impaired their osteogenic differentiation, and aggravated alveolar bone loss (36, 74). Low-intensity pulsed ultrasound (LIPUS) stimulation of stem cells from the apical papilla (SCAP) increased EV production and enriched miR-935, which promoted PDL-cell osteogenic differentiation and reduced inflammatory bone loss (66). More targeted cargo changes were achieved through genetic manipulation: P2X7R-transduced PDLSCs released EVs enriched in miR-3679-5p, miR-6747-5p, and miR-6515-5p that regulated GREM1 and restored osteogenic differentiation under inflammatory conditions (75), whereas Ckip-1-silenced M2-like macrophages produced let-7f-5p-enriched EVs that restored *Porphyromonas gingivalis*-impaired cementoblast mineralization through the Ckip-1–PGC-1α pathway (73).

EVs can also be loaded with exogenous therapeutic molecules after separation. Melatonin was introduced into M2-like macrophage EVs by ultrasonic loading, and the resulting preparation reduced endoplasmic-reticulum stress and apoptosis in inflammation-exposed hPDLCs while restoring osteogenic and cementogenesis-associated responses. Incorporation into GelMA enabled sustained local release and improved periodontal bone healing in rats with ligature-induced periodontitis (78). Post-separation loading thus added a defined therapeutic function to the EV preparation, while the hydrogel controlled its delivery within the periodontal site.

### 4.2 Surface engineering and targeting

Surface engineering aims to alter how EV preparations distribute within tissues and interact with recipient cells. This can be achieved indirectly by modifying donor cells to express a selected membrane protein that is subsequently incorporated into released EVs, or directly after separation through chemical conjugation, lipid insertion, or affinity-based attachment of peptides, antibodies, or other ligands (77). These strategies may enhance tissue retention or receptor-dependent cellular uptake. In a periodontal application, CXCR4-expressing 293T/17 cells were used to generate CXCR4-enriched EVs carrying miR-126. Compared with control EVs, the engineered EVs showed greater uptake by M1-like macrophages and promoted an anti-inflammatory macrophage response. Local administration in rat periodontitis reduced inflammatory cytokine expression, osteoclastogenesis, and alveolar bone resorption (79).

### 4.3 Biomaterial-assisted local delivery

Local delivery is particularly relevant to periodontal therapy because an EV preparation placed directly into an exposed defect may be rapidly dispersed by tissue fluid, degraded, or cleared from the treatment site. Biomaterials can increase local retention, regulate release, and provide a physical environment for tissue repair. Injectable hydrogels can conform to irregular defects and act as local depots, whereas porous collagen or mineral-based scaffolds can additionally provide structural support for cell migration and matrix deposition (80).

Collagen sponges, mineral scaffolds, chitosan and GelMA hydrogels, and microenvironment-responsive microparticles have been used to prolong EV exposure or provide structural support in periodontal models, with reported improvements in inflammatory control, bone preservation, vascularization, and attachment-related repair (32, 38, 58, 62, 78). These studies establish the feasibility of combining EV preparations with local carriers while also creating a composite intervention whose outcome may reflect the independent effects of the material and any additional therapeutic cargo.

Across these engineering strategies, the value of EV modification lies in addressing the dominant barrier to periodontal repair. Cargo engineering changes the biological signal being delivered, surface engineering alters interactions with the intended recipient cells, and biomaterial-assisted delivery regulates local retention while providing physical support within the defect. These requirements change during periodontal healing: control of inflammatory and osteoclastogenic activity creates the conditions for subsequent vascular, matrix, PDL, bone, and cementum-forming responses. An effective design therefore links what signal is delivered, which cells receive it, and where and for how long exposure is maintained. Cargo, surface, and carrier function as interacting design variables whose alignment can connect regulation of the periodontal microenvironment with reconstruction of the PDL–cementum–alveolar bone attachment apparatus.

## 5 Clinical translation challenges

Periodontal EV therapy remains largely at the proof-of-concept stage. Clinical translation depends on whether biologically active preparations can be manufactured reproducibly, delivered at a defined dose to periodontal defects, and evaluated for safety and efficacy using outcomes that distinguish control of periodontal destruction from reconstruction of the attachment apparatus.

### 5.1 Current clinical evidence

Human evidence currently comprises one registered early-phase study without posted results and two small uncontrolled clinical reports. The registered study NCT04270006 was designed as an open-label, single-group Early Phase 1 investigation of locally administered autologous adipose stem cell-derived exosomes as an adjunct to scaling and root planning in approximately 10 participants with advanced periodontitis. The record has not been updated since February 2020, its status is listed as unknown, and no results have been posted. The study therefore documents an early attempt to translate locally delivered EV treatment into periodontal care but provides no evaluable clinical outcome data.

Puletic et al. administered a single dose of a plasma-derived EV preparation into periodontal pockets in seven patients with stage I–III periodontitis and assessed clinical indices and gingival-crevicular-fluid cytokines six weeks later. Reductions in probing depth, bleeding, plaque accumulation, and several inflammatory cytokines were reported, together with a modest gain in clinical attachment level (81). The study provided initial evidence that local EV administration can be associated with short-term changes in periodontal inflammation and clinical status. Its uncontrolled before–after design, small sample, incompletely described concomitant periodontal treatment, and absence of structural imaging or histology prevent these changes from being interpreted as EV-specific periodontal regeneration.

A subsequent retrospective cohort included 13 patients with 23 advanced one- and two-wall periodontal osseous defects treated using a composite regenerative protocol consisting of a commercial placental EV preparation, anorganic bovine bone with collagen, and horizontal platelet-rich fibrin. At six months, the treated sites showed reductions in probing depth and gains in clinical attachment, with no adverse events reported during follow-up (82). These findings demonstrate the clinical feasibility of incorporating an EV preparation into periodontal reconstructive surgery, but the outcome represents the activity of the complete grafting protocol rather than that of the EV component alone. Taken together, the available human studies support further investigation of EVs as adjuncts for inflammatory control and defect healing, while controlled trials are needed to determine their contribution to durable reconstruction of periodontal attachment.

### 5.2 Product definition, manufacturing, and quality control

The biological activity of an EV preparation begins with the producing cell. Donor tissue, age, systemic health, medication exposure, passage number, culture density, oxygen tension, medium composition, three-dimensional culture, inflammatory conditioning, and harvest interval can alter EV yield and molecular composition (14, 83). These variables are especially relevant in periodontal applications because preconditioning can enrich therapeutic cargo, whereas diabetic, senescent, infectious, and persistently inflammatory environments can generate EVs with impaired or disease-promoting activity. Cellular source and conditioning history therefore form part of the product identity.

The separation process further defines the administered preparation. Differential ultracentrifugation, size-exclusion chromatography, tangential-flow filtration, precipitation, and affinity-based methods recover different proportions of vesicles and non-vesicular material. Differential ultracentrifugation is widely used at the research scale but presents limitations in recovery, aggregation, throughput, and operator dependence. Size-exclusion chromatography provides gentle separation from many soluble macromolecules, whereas tangential-flow filtration and chromatography-based workflows are more readily adapted to larger production volumes. The appropriate process depends on the starting material and the composition, concentration, and biological activity required for the final product (14, 23).

Reproducible manufacturing requires continuity between upstream cell production and downstream EV processing. Donor qualification, cell banking, passage limits, culture media, cell density, conditioning time, harvest criteria, separation, concentration, formulation, and filling influence the final EV preparation. When laboratory-scale culture is transferred to bioreactors or other scalable systems, changes in oxygenation, nutrient gradients, shear stress, and cell–matrix interactions can modify EV production and cargo. Process development must therefore preserve the biological activity identified during preclinical testing rather than focusing on particle yield alone (23, 84, 85).

Quality control links product composition with biological function. Particle concentration and size distribution, morphology, membrane integrity, EV-associated components, non-vesicular impurities, and batch consistency define the physicochemical profile, while sterility, endotoxin, mycoplasma, adventitious agents, and residual culture components determine microbiological and process safety. Engineered preparations additionally require measurement of cargo loading and residual loading or conjugation reagents. For EV–biomaterial constructs, characterization extends to EV recovery from the carrier, release behavior, material degradation, and retention of biological activity after release (14, 23, 85).

A mechanism-relevant potency assay is central to this framework. Depending on the intended periodontal indication, potency can be linked to regulation of inflammatory signaling, macrophage activity, endothelial responses, osteoclastogenesis, or the behavior of PDL, bone-lineage, and cementum-forming cells. The selected assay should provide a reproducible relationship between EV dose and the biological activity that supports the proposed therapeutic claim. Identity, purity, integrity, and particle quantity establish what has been manufactured; potency establishes whether the preparation retains its intended function (19, 85).

Formulation and storage complete the manufacturing process. Buffer composition, container material, temperature, storage duration, freeze–thaw exposure, transportation, and preparation at the clinical site can alter particle integrity and activity. Stability assessment should therefore follow both physicochemical attributes and potency across the proposed shelf life. MISEV2023 provides a foundation for transparent EV characterization and reporting, while clinical development additionally requires product-specific process controls, release specifications, stability limits, and comparability testing across manufactured batches (14, 23, 84, 85).

### 5.3 Dose, administration, biodistribution, and biosafety

EV dose has been expressed as particle number, total protein, preparation volume, and producer-cell equivalent, but each measure captures a different property of the preparation. Particle counts vary with the analytical platform and include particles that are not necessarily biologically active, while total protein is affected by soluble and co-isolated material. Producer-cell equivalents depend on culture conditions and harvest efficiency. A therapeutically meaningful dose therefore combines a defined amount of preparation with a measure of biological potency. Dose–response and time-course experiments can then identify the exposure required to initiate and sustain the intended periodontal response (20).

The administration route determines the relationship between the nominal dose and tissue exposure. Systemically administered EVs undergo rapid redistribution and are commonly detected in organs associated with the mononuclear phagocyte system, reducing the proportion that reaches periodontal tissues (21). Local administration places the preparation closer to gingival, PDL, vascular, and bone-remodeling cells and is therefore better aligned with periodontal defects. Retention within the treated site is nevertheless influenced by gingival crevicular fluid, saliva, enzymatic activity, vascular and lymphatic drainage, mechanical loading, and loss from the periodontal pocket. Hydrogels and scaffolds can prolong local exposure by controlling release and maintaining the preparation within the defect (80).

Biodistribution analysis must distinguish the administered EVs from the tracking system used to visualize them. Lipophilic fluorescent dyes can persist after EV degradation, transfer to other membranes, and form fluorescent particles independently of EVs (86). Reporter signals are therefore most informative when free-label controls and complementary molecular measurements are included. For locally delivered periodontal products, the critical distribution parameters are retention within the defect, penetration into relevant tissue compartments, uptake by the intended recipient cells, and exposure of distant organs.

Safety assessment follows the composition and route of the specific product. Local evaluation should examine inflammatory reactions, epithelial healing, root resorption, ankylosis, ectopic mineralization, and disruption of normal bone remodeling. Systemic assessment addresses organ toxicity, immunogenicity, coagulation, complement activation, and unintended vascular or immune effects. Introduced cargoes and surface modifications add the possibility of off-target activity and residual processing reagents, while biomaterial carriers introduce degradation products and material-associated inflammation. Repeated-dose and longer-term studies are particularly important for periodontal treatment because clinically relevant therapy may require sustained local release or repeated administration rather than a single exposure (19, 87).

### 5.4 Preclinical models and clinically meaningful outcomes

The preclinical model determines which component of therapeutic activity can be evaluated. Ligature- and pathogen-associated periodontitis models reproduce microbial challenge, inflammation, osteoclast activation, and alveolar bone loss and are suited to studying disease modification. Surgically created periodontal defects provide reproducible geometry and facilitate evaluation of reconstructive procedures. Calvarial and other non-periodontal bone-defect models establish osteogenic activity but lack the tooth root, cementum, PDL, gingival barrier, and periodontal microbial environment. Evidence from these models addresses different questions and should be interpreted within the anatomical and pathological conditions that each model reproduces (88).

Rodent studies provide efficient platforms for mechanistic analysis, dose exploration, and initial safety testing. Large-animal models add periodontal dimensions, defect morphology, surgical access, mechanical loading, and delivery volumes that more closely approximate clinical treatment. They are particularly useful for examining the handling and retention of EV–biomaterial constructs and the organization of newly formed periodontal tissues. Sequential use of mechanistic rodent models and anatomically relevant large-animal models can therefore connect biological activity with the practical requirements of periodontal reconstruction (88).

Outcome selection must distinguish suppression of ongoing destruction from regeneration of previously lost attachment. Reductions in inflammatory cytokines, osteoclast numbers, or alveolar bone loss demonstrate control of disease activity. Increased mineralized-tissue volume in a defect demonstrates bone formation. Periodontal regeneration requires the coordinated formation of new alveolar bone, new cementum on a previously affected root surface, and functionally oriented PDL fibers inserted between these mineralized tissues (4). Micro-computed tomography is valuable for measuring three-dimensional mineralized-tissue changes, but histology and histomorphometry are required to determine cementum formation, PDL organization, fiber orientation and insertion, epithelial downgrowth, vascularization, and tissue integration.

The temporal design of preclinical studies should follow the sequence of periodontal healing. Early observations capture EV retention, inflammatory regulation, vascular responses, and initial cell recruitment; later observations establish matrix organization, mineralized-tissue maturation, attachment formation, and durability. Randomization, blinded assessment, justified sample size, independent EV batches, and clinically relevant vehicle and carrier controls strengthen the connection between the administered product and the observed response. Comparison of engineered EVs with their unmodified counterparts further identifies the contribution of the engineering strategy. Together, model selection and endpoint hierarchy determine whether a study supports a claim of inflammatory control, preservation of existing attachment, repair of an individual compartment, or coordinated periodontal regeneration (14, 88).

### 5.5 Regulatory positioning and clinical-trial design

Therapeutic EV preparations are developed within existing medicinal-product frameworks, although their classification depends on jurisdiction, source, composition, engineering, formulation, route of administration, and intended use. In the United States, the FDA has stated that exosome products intended to treat disease are generally regulated as drugs and biological products and are subject to premarket review. In the European Union, EV preparations are not automatically classified as advanced therapy medicinal products; classification depends on product composition and mechanism, and recombinant nucleic-acid cargo can introduce gene-therapy considerations. EV–biomaterial constructs can additionally involve combination-product or device-related requirements. Regulatory classification therefore develops from the properties and clinical claim of the final product rather than from the general designation “EV” or “exosome” (87).

A candidate entering first-in-human investigation requires continuity between the product used in pivotal preclinical studies and the material administered clinically. The manufacturing process, release specifications, potency assay, formulation, storage conditions, and stability profile define this continuity. Major changes in cellular source, culture scale, separation, cargo engineering, or carrier composition require analytical and functional comparability because they can change the biological activity of the preparation (19, 87).

The clinical indication determines trial design. An EV product intended to control residual inflammation after nonsurgical therapy requires different participants and endpoints from a construct intended to regenerate intrabony or furcation defects. In periodontal regenerative applications, EV therapy should be positioned as an adjunct to biofilm control, debridement, and established regenerative procedures. Eligibility criteria should define disease activity, defect morphology, oral-hygiene status, smoking, metabolic disease, medication exposure, and other factors that influence healing. When EVs are delivered through a scaffold or hydrogel, the comparator should receive the same periodontal procedure and carrier without EVs, allowing the incremental activity of the EV component to be determined.

Early-phase trials should establish safety, tolerability, delivery feasibility, dose range, local and systemic exposure, and pharmacodynamic activity. Randomization, allocation concealment, blinded periodontal examination, standardized supportive care, and documentation of the administered EV batch become increasingly important as development progresses toward efficacy testing. Multicenter studies additionally require calibration of clinical measurements and standardized product storage, preparation, and administration across treatment sites (19).

Clinical endpoints should correspond to the intended therapeutic effect. Probing-depth reduction, clinical attachment-level gain, bleeding control, radiographic defect fill, tooth mobility, tooth retention, adverse events, and patient-reported pain, function, and oral-health-related quality of life capture complementary dimensions of clinical benefit. Inflammatory and bone-remodeling biomarkers can demonstrate biological activity but do not replace clinical outcomes. Longer follow-up is needed to determine whether attachment gains remain stable, inflammation recurs, and reconstructed tissues mature without late root resorption, ankylosis, or ectopic mineralization. The central clinical distinction is therefore between control of periodontal disease, improvement of defect healing, and regeneration of the complete periodontal attachment apparatus.

## 6 Discussion and future perspectives

The available evidence indicates that EVs influence periodontal repair by reshaping interactions among immune, epithelial, stromal, vascular, and mineralized-tissue compartments. Regulation of inflammatory and osteoclastogenic activity can reduce the destructive pressure imposed on periodontal tissues, while effects on barrier cells, endothelial cells, PDL cells, osteoblast-lineage cells, and cementoblasts influence the subsequent repair response. The regenerative significance of these activities lies in their spatial and temporal coordination, because recovery of an individual cell population or tissue compartment does not by itself reconstruct the gingiva–PDL–cementum–alveolar bone complex.

Evidence is most consistent for regulation of inflammatory signaling, macrophage activity, osteoclastogenesis, alveolar bone loss, and early cellular responses associated with wound healing, angiogenesis, and osteogenesis. A smaller group of studies has examined PDL organization and cementoblast activity, and only limited *in vivo* evidence demonstrates newly organized PDL fibers associated with mineralized-tissue formation. The available human studies report short-term improvements in inflammatory and clinical periodontal indices but do not establish structural regeneration of the attachment apparatus. Current evidence therefore supports EVs more strongly as regulators of the destructive periodontal environment and early repair than as independently validated inducers of complete periodontal regeneration.

Differences among reported EV effects can be understood through three interacting determinants: the biological signal produced by the donor cell, the state of the recipient periodontal environment, and the magnitude and duration of local exposure. Donor-cell conditioning changes EV cargo and surface composition, but the resulting activity depends on how recipient cells respond within inflammatory, metabolic, and microbial conditions. Delivery then determines whether this interaction is sufficiently sustained to influence tissue repair. From this perspective, cellular origin alone does not define therapeutic activity. A process-defined EV preparation must instead be considered together with the periodontal condition in which it is applied. Engineering is most informative when it corrects an identified limitation in this relationship by enriching a relevant signal, directing uptake toward a specified recipient population, or maintaining an effective local exposure.

Future studies should move beyond demonstrating isolated changes in cytokine expression, cell proliferation, mineralization markers, or bone volume. The central question is whether EV-mediated regulation of inflammation is followed by vascular and matrix stabilization, recovery of tissue-specific cell function, and formation of a durable periodontal attachment. This sequence requires evaluation in models that retain the inflammatory and microbial features of periodontitis, together with longitudinal assessment of gingival integrity, PDL organization, cementum formation, alveolar bone remodeling, and their structural integration. Mechanistic experiments should identify which EV-associated signals initiate these transitions and whether interruption of the proposed pathway alters the integrated tissue response.

Clinically, EV-based interventions are best positioned as adjuncts to effective infection control and established periodontal regenerative procedures. Their principal value may lie in converting a persistently destructive defect into an environment that can support healing and attachment reconstruction. Translation should therefore focus on defined EV preparations developed for specific periodontal indications rather than on EVs as a universal therapeutic category. The decisive evidence will be reproducible improvement in clinically meaningful outcomes accompanied by stable reconstruction of the periodontal attachment apparatus, not the accumulation of favorable but disconnected cellular responses.
